# DNA‐Mediated Protein Shuttling between Coacervate‐Based Artificial Cells

**DOI:** 10.1002/anie.202115041

**Published:** 2022-02-26

**Authors:** Tsuyoshi Mashima, Marleen H. M. E. van Stevendaal, Femke R. A. Cornelissens, Alexander F. Mason, Bas J. H. M. Rosier, Wiggert J. Altenburg, Koji Oohora, Shota Hirayama, Takashi Hayashi, Jan C. M. van Hest, Luc Brunsveld

**Affiliations:** ^1^ Laboratory of Chemical Biology Department of Biomedical Engineering and Institute for Complex Molecular Systems Eindhoven University of Technology P.O. Box 513 5600MB Eindhoven The Netherlands; ^2^ Department of Applied Chemistry Graduate School of Engineering Osaka University Suita 565-0871 Japan

**Keywords:** Coacervates, DNA, Proteins, Supramolecular Signalling, Synthetic Cells

## Abstract

The regulation of protein uptake and secretion is crucial for (inter)cellular signaling. Mimicking these molecular events is essential when engineering synthetic cellular systems. A first step towards achieving this goal is obtaining control over the uptake and release of proteins from synthetic cells in response to an external trigger. Herein, we have developed an artificial cell that sequesters and releases proteinaceous cargo upon addition of a coded chemical signal: single‐stranded DNA oligos (ssDNA) were employed to independently control the localization of a set of three different ssDNA‐modified proteins. The molecular coded signal allows for multiple iterations of triggered uptake and release, regulation of the amount and rate of protein release and the sequential release of the three different proteins. This signaling concept was furthermore used to directionally transfer a protein between two artificial cell populations, providing novel directions for engineering lifelike communication pathways inside higher order (proto)cellular structures.

## Graphical abstract

Proteins can be sequestered and released from artificial cells by attachment of DNA strands and using DNA strand displacement reactions. The DNA elements use a combined regulation mechanism of electrostatic interactions and specific sequence recognition. This provides control over rate of protein release, amount of protein released, and the multiplexity of release.

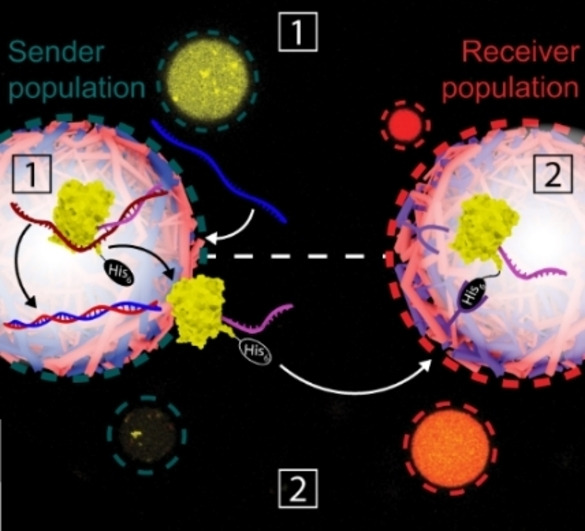

Inspired by natural communication processes, scientists have succeeded in implementing signaling networks in various bottom‐up synthetic cell designs in order to mimic intra‐ and intercellular signaling, and study such basic signaling events in a highly controlled manner.[^1^, ^2^, ^3^] These designs are for example driven by catalytic conversion,[^4^, ^5^, ^6^, ^7^, ^8^] genetic circuits,[^9^, ^10^] or implementation of membrane channels,[^11^, ^12^] which precede the diffusion of small signaling molecules from artificial cells to their environment. Though less abundantly studied, especially when considering their importance in natural cellular communication, proteins and peptides have also been used as signaling molecules in synthetic communication pathways.^[13]^ Their controlled release has been achieved using vesicle fusion events,^[14]^ enzyme mediated cleavage of proteins from the artificial cell matrix^[15]^ or cell free protein synthesis.[^16^, ^17^] While these approaches can be used to effectively regulate whether a protein is localized intra‐ or extracellularly, they often lack the possibility to precisely trigger and control both the amount and the nature of the protein released. Such multiplex control would represent an important step towards engineering more intricate intercellular communication systems.[^9^, ^18^]

Coacervates have been increasingly studied as bottom‐up artificial cell platforms, owing to their crowded nature and their potential to act as framework on which semi‐permeable membranes can be assembled.[^6^, ^19^, ^20^, ^21^, ^22^] Moreover, in nature, analogous liquid‐liquid phase separated compartments have been found to localize macromolecules and thus cellular processes and as such, they have the innate ability to sequester proteins.[^23^, ^24^] Only a few coacervate‐based artificial cell examples exist where specific interactions between the protein and coacervate matrix were employed.[^15^, ^25^, ^26^] The vast majority of protein sequestration has been reported via preferential partitioning, often based on electrostatic interactions between the protein and the coacervate matrix.[^27^, ^28^, ^29^, ^30^, ^31^] Sequestration via such interactions is generally easy to achieve, but difficult to control after initial loading. Herein we exploit DNA strand displacement reactions to regulate the sequestration and release of proteins from coacervate‐based artificial cells. While artificial, this molecular regulation concept allows for control over the rate and quantity of protein release, multiplexing of protein release, and exchange of proteins between artificial cell populations.

We have previously reported a robust artificial cell platform comprising charged amylose‐based coacervate droplets and a stabilizing semi‐permeable triblock copolymer poly(ethylene glycol)‐poly‐(caprolactone‐*gradient*‐trimethylene carbonate)‐poly‐(glutamic acid) (Figure 1A and B).[^21^, ^32^] The coacervates are constructed with an excess positive charge, allowing the sequestration of negatively charged proteins, while excluding neutral proteins.[^15^, ^33^] By modifying neutrally charged proteins with a short oligonucleotide tag, single‐stranded DNA oligos (ssDNA, uptake (UPT) strands) are envisioned to be reversibly attached to equip the proteins with enough anionic charge to be sequestered (Figure 1C, D). Furthermore, following removal of this ssDNA, the subsequent exclusion of the proteins from the artificial cell should be enabled (Figure 1E).

**Figure 1:**
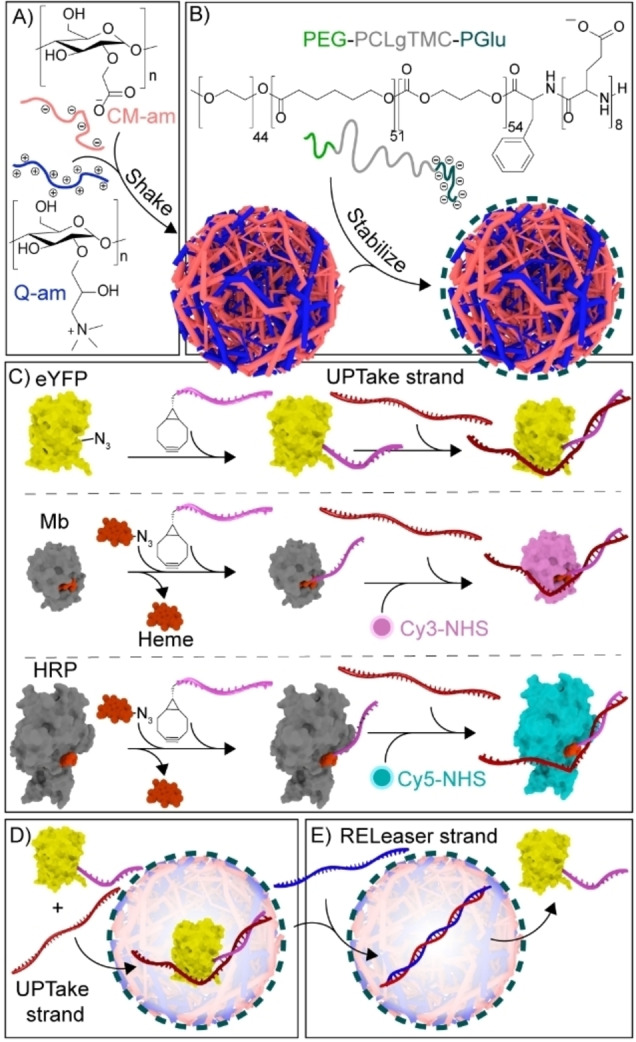
Schematic illustrating protein recruitment and release from coacervate‐based artificial cells using DNA strand displacement. A) Liquid–liquid phase separation of oppositely charged cationic quaternized amylose (Q‐am) and anionic carboxymethyl‐amylose (CM‐am) into microdroplets. B) Hierarchical self‐assembly of the terpolymer (PEG‐PCLgTMC‐PGlu) on the droplet surface. C) Preparation of protein–DNA conjugates via BCN–azide click chemistry. The UPT strand was attached to connected‐DNA on proteins. Cy3 and Cy5 dyes were covalently introduced to Mb and HRP, respectively, before introduction of UPT strand. D) Exemplary sequestration of eYFP in the coacervate core following hybridization to a complementary DNA uptake (UPT) strand. E) Release of eYFP from the coacervate matrix triggered by the addition of a displacing releaser (REL) DNA strand.

In order to create an attachment site for the UPT strand, short 12 nucleotide (nt) ssDNAs were site‐specifically conjugated via their 5′ end to three different proteins to act as a complementary, sequence‐selective and responsive handle; enhanced Yellow Fluorescent Protein (eYFP), Myoglobin (Mb) and Horseradish peroxidase (HRP) were modified via a strain‐promoted azide‐alkyne cycloaddition (Figure 1C, S1 and S2). For eYFP, the 12nt‐ssDNA was directly conjugated to an azide‐modified eYFP. For Mb and HRP, an azide–modified heme, as an artificial cofactor, was conjugated to the 12nt‐ssDNA. For visualization purposes, Mb and HRP were additionally labeled with Cy3 and Cy5 dyes, respectively.

Conjugation of the 12nt‐handle alone did not trigger protein partitioning in the coacervate phase, as determined by confocal microscopy (Figure 2A). However, hybridization of a partially complementary 20+12nt UPT strand to the 12nt‐ssDNA‐protein conjugates was sufficient to trigger their sequestration within 30 minutes (Figure 2A). Interestingly, this was only the case when the 20nt non‐complementary part of the UPT strand was located at the 5′‐end, but not at the 3′‐end of the 12nt‐handle on Mb (Figure S3). These results suggest a sequestration mechanism where not only the anionic charge of the ssDNA, but also an interaction between the ssDNA and the protein surface influences its efficiency.

**Figure 2:**
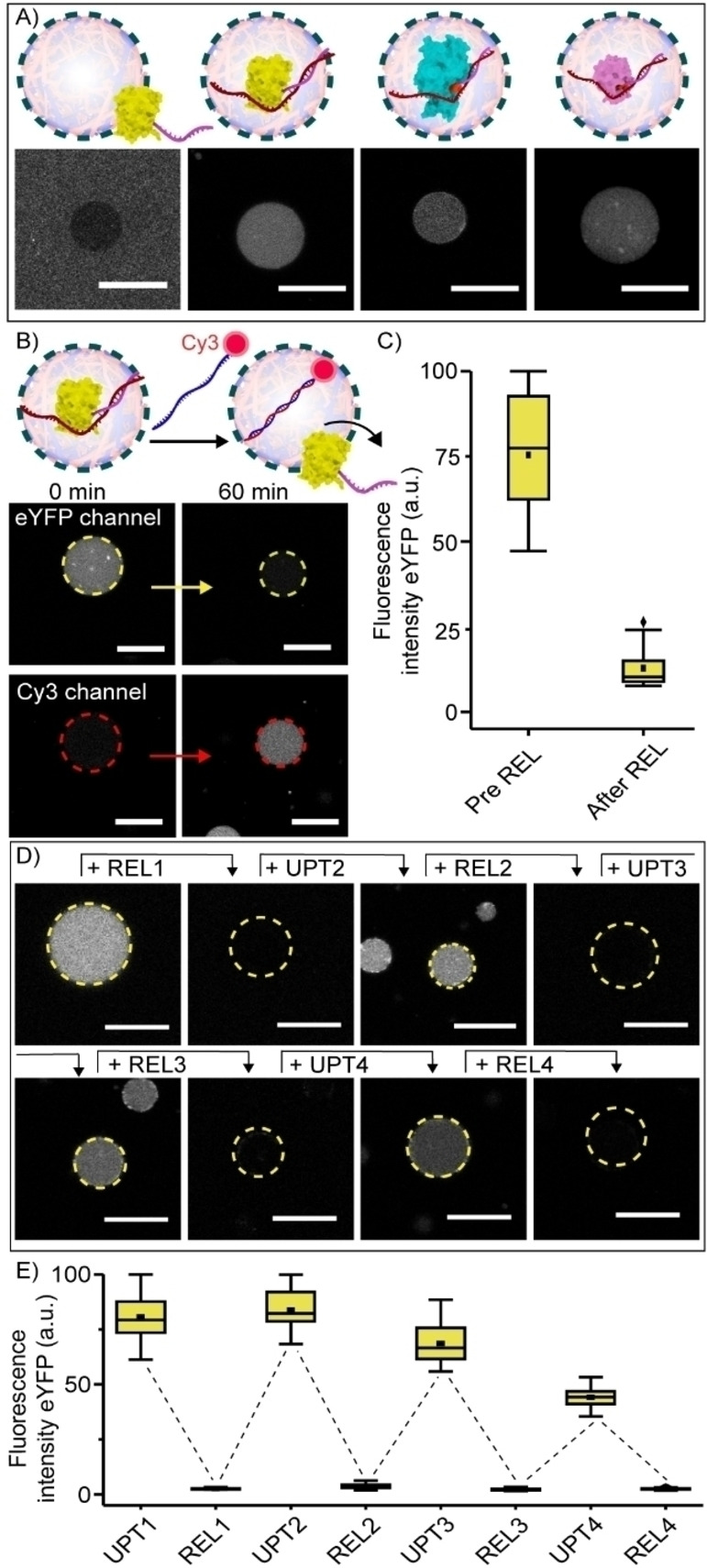
Dynamic uptake and release of protein inside coacervate artificial cells using DNA strand displacement. A) Schematic and confocal images of the partitioning of 12nt‐ssDNA‐eYFP, 12nt‐ssDNA‐HRP and 12nt‐ssDNA‐Mb (100 nM each) inside the coacervate core following DNA hybridization to a complementary UPT strand. Omitting UPT prevents uptake. Incubation time was 30 minutes at 4 °C. B) Schematic and confocal images of 12nt‐ssDNA‐eYFP (100 nM) release from coacervates triggered by the addition of a Cy3‐labeled release strand (REL, 120 nM). Different time points show different artificial cells. After adding the REL strand, the solution was incubated for 60 minutes at room temperature. C) Box‐plots displaying the quantified fluorescence intensity of eYFP inside the coacervate core. D) Representative confocal images of the cycling of eYFP uptake and release from coacervates following the consecutive addition of UPT and REL strands. Concentrations of 12nt‐ssDNA‐eYFP and UPT strand were 100 nM at UPT1 step. 120 nM REL strand was added at REL1 step, and then 500 nM UPT and 600 nM REL strands were added at each uptaking and releasing steps, respectively. The incubation time at each step was 15 minutes. Different time points show different artificial cells. E) Box‐plots displaying the quantified fluorescence intensity of eYFP inside coacervates during the cycle. All experiments were performed in PBS containing 5 mM MgCl_2_, pH 7.4, ionic strength (*I*)=185 mM. Fluorescence intensity was determined inside the core of≥15 coacervates. ▪ represents the mean, ♦ represents outliers. Scale bars represent 20 μm.

Sequestration of eYFP, studied as an example, was reversible. This was demonstrated by the addition of a Cy3 labeled 20+12nt releaser strand (REL, Figure 2B), which was fully complementary to the uptake strand. The REL strands were taken up homogeneously inside the artificial cells, bound to the UPT and displaced 12nt‐ssDNA‐eYFP, triggering its release (Figure 2B and C). When the REL strand was omitted, eYFP remained inside the artificial cells (Figure S4). To test the specificity of this process, release was also studied with a 10‐fold excess of mismatched DNA mixed in with REL. The presence of the mismatched DNA did not impair release of 12nt‐ssDNA‐eYFP (Figure S5). In addition, increasing the concentration NaCl in the sample with approximately 25 %, decreased uptake efficiency but did not influence 12nt‐ssDNA‐eYFP release, demonstrating the robustness of the system in conditions that can reduce electrostatic interactions (Figure S6).

The uptake and release process could be cycled for at least four times (Figure 2D and E). Interestingly, in subsequent rounds (UPT3‐4) the sequestration efficiency decreased somewhat, while the releasing efficiency was not affected. This might indicate that the accumulation of ssDNA in the coacervate decreases its net positive charge, in turn reducing the tendency to sequester the DNA‐eYFP conjugates. In fact, the net positive charge of the system was estimated to be approximately 2/3 of its initial value after the addition of UPT3 (Table S6).

The ability to achieve DNA‐triggered control over the amount, speed, and type of protein release was subsequently evaluated. First, the stepwise release of 12nt‐ssDNA‐eYFP was shown by tuning the addition of the REL strands. Specifically, 1 equivalent of 12nt‐ssDNA‐eYFP was released in two steps, as a consequence of the 2 times addition of 0.5 equivalents REL strand (Figure 3A).

**Figure 3:**
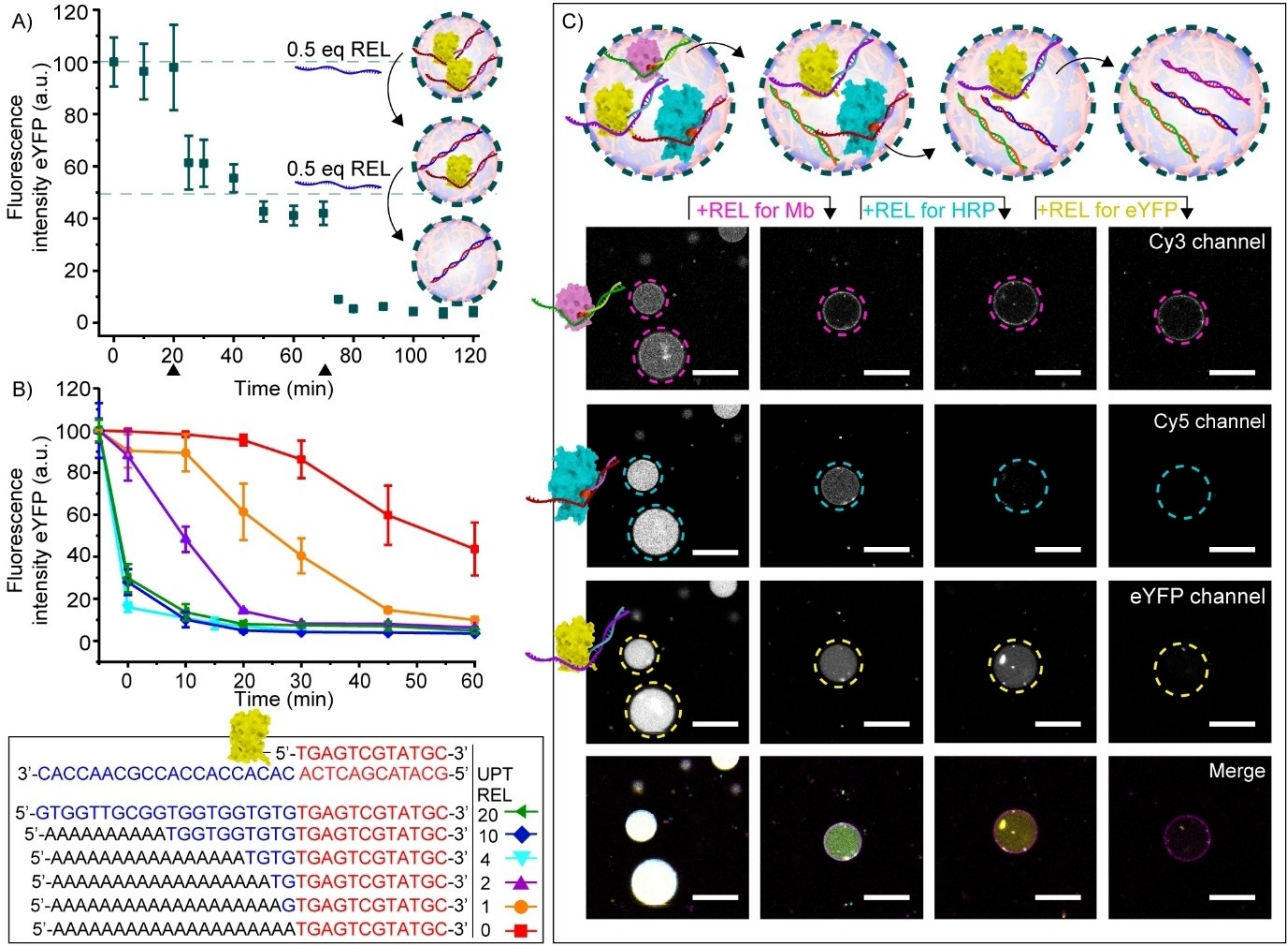
Multiplex control over protein release from coacervate‐based artificial cells. A) Schematic and quantification of the release of 1 equivalent eYFP (100 nM) from coacervate‐based artificial cells following two times addition of 0.5 equivalents REL strand (50 nM) at 20 and 70 minutes (black arrowheads). Graph depicts the decrease in eYFP fluorescence intensity inside coacervates over time. B) Dependence of REL strand complementarity on releasing speed of eYFP. Graph depicts the decrease in fluorescence intensity inside coacervates over time, following the addition of REL strands with complementary bases varying between 12+0 nt (0) and 12+20 nt (20) at room temperature. Fluorescence intensity was determined inside the core of ≥5 coacervates. Error bars represent standard deviation. For uptaking eYFP with each UPT strands, all samples were incubated over 30 minutes before adding each REL strands at 4 °C. C) Schematic and confocal images showing the selective step‐wise release of Mb, HRP and eYFP following the consecutive addition of corresponding REL strands. After adding each REL strand, the solutions were incubated for 30 minutes at room temperature. All experiments were performed in PBS containing 5 mM MgCl_2_, pH 7.4, ionic strength (*I*)=185 mM. Different time points represent different artificial cells. Scale bars represent 20 μm.

The release speed was shown to be dependent on the sequence complementarity of the REL and UPT strand. A fully complementary REL strand (REL 20, 100 % complementary to UPT) resulted in complete protein release within 10 minutes (Figure 3B and Figure S7). In contrast, when the 20nt part of the REL strand was replaced with non‐complementary adenosines (REL 0, only 37.5 % complementary to UPT), the speed of eYFP release declined, but reached completion after 24 hours (Figure 3B and S8). Although the protein releasing speed was also dependent on other factors, such as the precise method of REL strand addition and content mixing, a clear trend was shown with the number of complementary bases being inversely correlated with the releasing speed (Figure 3B and S7).

In an attempt to replicate the competitive environment in natural cells where many different signaling molecules are present, two closely related releasing strands were designed (REL 2 and REL 2* (Figure S9). Upon addition to eYFP loaded coacervates, only the strand with a 100 % complementarity to the handle sequence was able to release eYFP.

The DNA strand displacement concept provides a simplified, yet important and highly challenging first step, towards mimicking the cellular multiplex control over protein release.[^34^, ^35^, ^36^] To demonstrate this concept, different orthogonal combinations of 12nt‐handle—UPT—REL strands were designed (Table S1–S3). After confirming the selectivity of these designs (Figure S10), eYFP, Mb and HRP were each modified with a different 12nt‐handle. Following hybridization with their corresponding UPT strand, the three proteins were co‐sequestered inside coacervates. First, the REL strand for Mb was added, to selectively hybridize with the Mb UPT strand, thus displacing the 12nt‐ssDNA‐Mb. This indeed resulted in the selective release of Mb, with the majority of HRP and eYFP remaining in the coacervates (Figure 3C and S11). Interestingly, the 12nt‐ssDNA‐Mb conjugates subsequently formed a corona around the coacervate droplets, an interaction with the terpolymer not observed for the other two proteins. Subsequently, after addition of REL for HRP, only eYFP remained inside the artificial cells, and finally, after addition of the REL strand for eYFP, all three proteins were released from the artificial cells. When the REL strands were omitted, all proteins remained inside the coacervate core (Figure S12).

Finally, the DNA‐triggered transport of a protein from one population of artificial cells to another was shown. This was achieved by the DNA‐triggered release of eYFP which contained a His‐tag, known to specifically bind within artificial cells containing nitrilotriacetic acid (NTA) amylose.^[15]^ A “sender” artificial cell population was equipped with 12nt‐ssDNA‐eYFP‐His and its corresponding UPT strand (Figure 4A). In a second, receiver, population the coacervate matrix was equipped with NTA‐amylose (Figure 4A, B). Upon addition of the REL strand, the 12nt‐ssDNA‐eYFP‐His was released from the sender population and rapidly sequestered by the receiver population (Figure 4C, D and Video S1). Omitting the REL strand prohibited signal transduction. (Figure S13).

**Figure 4:**
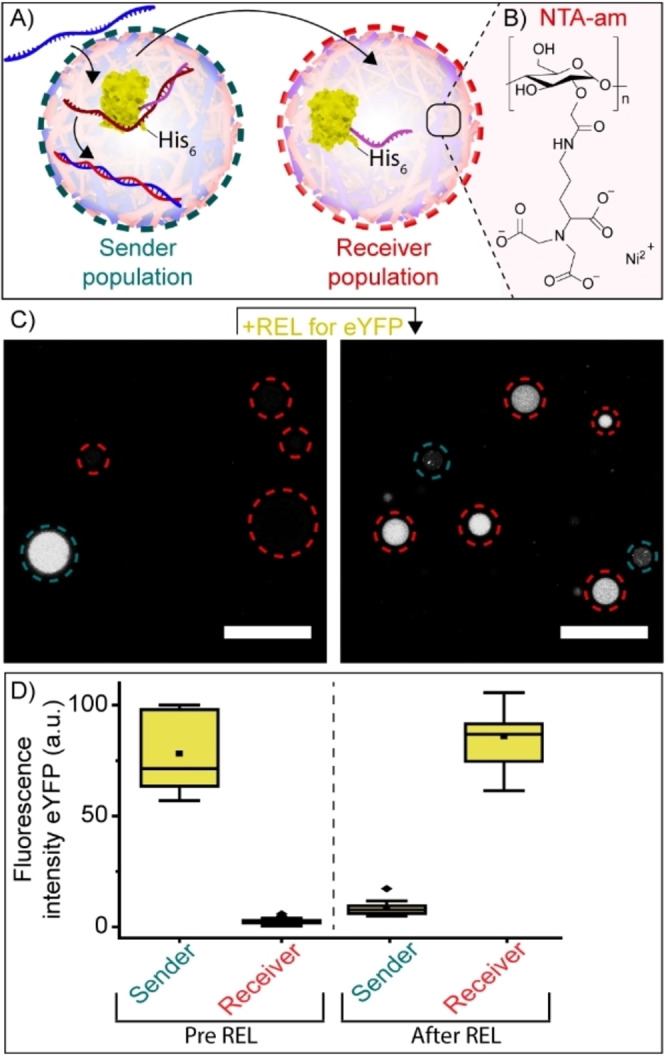
Protein shuttling between two different coacervate‐based artificial cell populations. A) Schematic showing exchange between two artificial cell populations. The sender population was loaded with eYFP and UPT strand, the receiver population was equipped with NTA‐amylose and Ni^2+^. Upon addition of REL strands, His‐tagged eYFP was released from the sender population and incorporated by the receiver population. B) Structure of NTA‐amylose. C) Confocal images showing the release and subsequent uptake of eYFP (100 nM) from the sender population (blue) to the receiver population (red). After adding the REL strand, the solution was incubated for 60 minutes at room temperature. The experiment was performed in PBS containing 5 mM MgCl_2_ and 3.75 μM NiCl_2_, pH 7.4, *I*=185 mM. D) Box plots displaying the quantified fluorescence intensity of eYFP inside the sender and receiver artificial cell population before and after addition of the REL strand. Fluorescence intensity was determined inside the core of ≥15 coacervates. ▪ represents the mean, ♦ represents outliers. Scale bars represent 20 μm.

Here we have reported the controlled sequestration and release of proteins from artificial cells using DNA strand displacement reactions. This synthetic analogue to protein secretion provides an unrivalled level of programmable control over the transport of proteins across synthetic cell membranes. The DNA elements enable the regulation of electrostatic interactions between proteinaceous cargo and the coacervate core. Moreover, the specificity afforded by the DNA elements provides a fine degree of control over parameters such as the rate of release, the stoichiometric amount of material released, and the multiplexity of release. These results demonstrate that this coacervate‐based artificial cell system represents a versatile platform to establish protein‐based signal transduction for studying more complex, lifelike chemical communication pathways in synthetic cells.

## Conflict of interest

The authors declare no conflict of interest.

## Supporting information

As a service to our authors and readers, this journal provides supporting information supplied by the authors. Such materials are peer reviewed and may be re‐organized for online delivery, but are not copy‐edited or typeset. Technical support issues arising from supporting information (other than missing files) should be addressed to the authors.

Supporting Information

Supporting Information

## Data Availability

The data that support the findings of this study are available in the supplementary material of this article.
