# Supplementary material for: DNA‐Mediated Protein Shuttling between Coacervate‐Based Artificial Cells
Source: Angew Chem Int Ed Engl. 2022 Feb 26;61(17):e202115041. doi: 10.1002/anie.202115041 (PMC9303767; doi:10.1002/anie.202115041)
Supplement: Supplementary file 1 — Supporting Information [file ANIE-61-0-s001.pdf]

Angewandte Chemie
Eine Zeitschrift der Gesellschaft Deutscher Chemiker

Supporting Information

DNA-Mediated Protein Shuttling between Coacervate-Based Artificial Cells

T. Mashima, M. H. M. E. van Stevendaal, F. R. A. Cornelissens, A. F. Mason, B. J. H. M. Rosier, W. J. Altenburg, K. Oohora, S. Hirayama, T. Hayashi, J. C. M. van Hest, L. Brunsveld**

Materials and methods

Materials and instruments

Solvents and reagents were obtained from commercial sources and used without further purification unless otherwise stated. Porphyrin **1**, protoporphyrin IX mono *t*-butyl ester, was synthesized according to the procedure reported in the literature.[1] Each compound, mono-substituted protoporphyrin IX or protoheme IX, **1**-**3** and **Heme-azide**, also forms a similar mixture of two regioisomers. All amine-modified and dye-labeled ssDNA strands were obtained HPLC-pure from IDT Integrated DNA Technologies, whereas all unlabeled DNA strands were obtained purified by using a desalting column. Upon arrival, all DNA strands were dissolved in milliQ in DNA Lo binding tubes (Eppendorf) to reach a final concentration of 0.1-1 mM. Myoglobin (M0630) and Horseradish Peroxidase (77332) were purchased from Sigma-Aldrich. ESI-TOF MS analyses were performed with a micrOTOF-II mass spectrometer (Bruker). ^{1}H NMR spectra were collected on an AVANCE III HD (400 MHz) NMR spectrometer (Bruker). The ^{1}H NMR chemical shift values are reported in ppm relative to a residual solvent peak.

Table S1. ssDNA sequences connected to proteins

Handle ssDNA strand	DNA sequence	Tm
Handle a	5'-AmMC6-TGAGTCGTATGC-3'	45.3 ˚C (vs UPT A-20)
Handle b	5'-AmMC6-GACGGAACTGAC-3'	49.3 ˚C (vs UPT B-20)
Handle c	5'-AmMC6-GTGATGCAGGAG-3'	48.4 ˚C (vs UPT C-20)

Melting temperatures were calculated with DINAMelt.[2] Calculation conditions: [ssDNA] = 100 nM, 140 mM Na^+, 5mM Mg^{2+}.

Table S2. ssDNA sequences of uptake strands (UPT)

Uptake ssDNA strand	DNA sequence	Tm
UPT A-10	5'-GCATACGACTCACACCACCGCA-3'	64.0 ˚C (vs REL A-20)
UPT A-20	5'-GCATACGACTCACACACCACCACCGCAACCAC-3'	78.2 ˚C (vs REL A-20)
UPT A-40	5'-GCATACGACTCACAACCCACAACACACCACCACCGCAACCACCCACCACCAA-3'	72.2 ˚C (vs REL A-20)
UPT B-20	5'-GTCAGTTCCGTCCAACTAAACAAATAACATAC-3'	69.4 ˚C (vs REL B-20)
UPT C-20	5'-CTCCTGCATCACATAAGAATAATACAACAACA-3'	67.8 ˚C (vs REL C-20)

Melting temperatures were calculated with DINAMelt. Calculation conditions: [ssDNA] = 100 nM, 140 mM Na^+, 5mM Mg^{2+}.

Table S3. ssDNA sequences of releaser strands (REL)

Releaser ssDNA strand	DNA sequence	Tm
REL A-20	5'-GTGGTTGCGGTGGTGGTGTGTGAGTCGTATGC-Cy3-3'	78.2 ˚C (vs UPT A-20)
REL A-10	5'-AAAAAAAAAATGGTGGTGTGTGAGTCGTATGC-Cy3-3'	68.5 ˚C (vs UPT A-20)
REL A-4	5'-AAAAAAAAAAAAAAAATGTGTGAGTCGTATGC-Cy3-3'	57.4 ˚C (vs UPT A-20)
REL A-2	5'-AAAAAAAAAAAAAAAAAATGTGAGTCGTATGC-Cy3-3'	52.9 ˚C (vs UPT A-20)
REL A-2*	5'-AAAAAAAAAAAAAAAAAATGAGAGTCGTATGG-Cy3-3'	43.9 ˚C (vs UPT A-20)
REL A-1	5'-AAAAAAAAAAAAAAAAAAAGTGAGTCGTATGC-Cy3-3'	50.5 ˚C (vs UPT A-20)
REL A-0	5'-AAAAAAAAAAAAAAAAAAAATGAGTCGTATGC-Cy3-3'	46.8 ˚C (vs UPT A-20)
REL B-20	5'-GTATGTTATTTGTTTAGTTGGACGGAACTGAC-Cy3-3'	69.4 ˚C (vs UPT B-20)
REL C-20	5'-TGTTGTTGTATTATTCTTATGTGATGCAGGAG-Cy3-3'	67.8 ˚C (vs UPT C-20)

Melting temperatures were calculated with DINAMelt. Calculation conditions: [ssDNA] = 100 nM, 140 mM Na^+, 5mM Mg^{2+}.

Table S4. sequence and parameters of eYFP

Protein	Sequence	Mass (kDa):	Theoretical pI:
eYFP	MVSKGEELFTGVVPILVELDGDVNGHKFSVSGEGEGDATYGKLTLKFICTT GKLPVPWPTLVTTFGYGLQCFARYPDHMKQHDFFKSAMPEGYVQERTIFF KDDGNYKTRAEVKFEGDTLVNRIELKGIDFKEDGNILGHKLEYNYNSHNVXI MADKQKNGIKVNFKIRHNIEDGSVQLADHYQQNTPIGDGPVLLPDNHYLSY QSALSKDPNEKRDHMVLLEFVTAAGITLGMDELYKKAAALELPETGGHHH HHH	28.9	5.9

X (Red): non-canonical amino acid p-azido-phenylalanine, Green: C-terminal hexahistidine tag.

Table S5. Parameters of commercial proteins Mb and HRP

Protein	Catalog number Sigma-Aldrich	Mass (kDa) :	Theoretical pI:
Mb	M0630	17.6	7.3 (major), 6.8 (minor)
HRP	77332	40.0	Native

Methods

Expression and purification of p-azido-phenylalanine (Y151pAzF) eYFP using amber stop codon suppression

The gene for enhanced yellow fluorescent protein (eYFP) was available in a pET28a expression vector with a C-terminal hexahistidine tag, and kindly provided by Remco Arts and Maarten Merkx. The native tyrosine at position 151 was mutated to encode for an amber stop codon for incorporation of *p*-azidophenylalanine, using the QuikChange Lightning Multi Site-Directed Mutagenesis kit (Agilent), according to the manufacturer's instructions (using primer 5'-ACTACAACAGCCACAACGTCTAGATCATGGCCGAC-3') The plasmid encoding for eYFP-Y151pAzF was co-transformed into *E. coli* BL21(DE3) competent bacteria together with a pEVOL plasmid containing an engineered orthogonal amino acyl tRNAse/tRNA pair from M. janaschii (a kind gift from Peter Schultz, Addgene plasmid #31186).[3],[4] The bacteria were cultured at 37°C in 200 mL LB medium, 25 µg/mL kanamycin and 25 µg/mL chloramphenicol. Protein expression was induced at OD_{600}=0.6 by addition of 1 mM β-D-1-thiogalactopyranoside and 0.02% (w/v) arabinose. Simultaneously, *p*-azido-L -phenylalanine was added directly to the culture medium at a final concentration of 1 mM. Expression was carried out in the dark for ~16 h at 30°C. Cells were harvested by centrifugation at 10,000 g for 10 min at 4°C and lysed by resuspending the pellet in BugBuster (5 mL/g pellet, Merck) supplemented with Benzonase nuclease (25 U per 10 mL buffer) for 45 min on a shaking table at room temperature. The soluble fraction (cleared lysate) containing protein was collected by centrifugation at 40,000 g for 30 min at 4°C. Purification was performed by Ni^{2+}-affinity chromatography using the C-terminal hexahistidine tag. This also removed protein fragments truncated at position 151, which is typically seen as a result of unsuccessful amber codon suppression. The cleared lysate was loaded on a Ni-charged column (His-Bind® Resin, Novagen), washed with wash buffer (1×PBS, 370 mM NaCl, 10% (v/v) glycerol, 20 mM imidazole, pH 7.4), and eluted with elution buffer (1×PBS, 370 mM NaCl, 10% (v/v) glycerol, 250 mM imidazole, pH 7.4). Buffer of the elution fractions was exchanged to storage buffer (100 mM Tris, 150 mM NaCl,1 mM EDTA, pH 8.0) with a PD10 desalting column (GE health care) and concentrated using Amicon 10 kDa MWCO centrifugal filters (Merck Millipore) to a final concentration of 1.85 mg/mL (60 µM), and then snap frozen in liquid nitrogen and stored in 500 µL aliquots at -80°C. The concentration of eYFP was determined by measuring the absorption at 513 nm assuming an extinction coefficient of 8.4×10^4 M^{-1} cm^{-1}.[4]

Preparation and characterization of eYFP-ssDNA conjugates

Preparation of BCN-labeled ssDNA

Functionalization of 5'-amine modified ssDNA with strained cyclooctynes was performed on 100µL scale by adding 4 mM (1R,8S,9s)-bicyclo[6.1.0]non-4-yn-9-ylmethyl N-succinimidyl carbonate (BCN-NHS, 10 mM stock solution in dry DMSO) to 200 µM of 5'-amine modified ssDNA in 1xPBS (pH 7.4), and incubating for 2 h at RT under continuous shaking at 800 rpm. Excess BCN-NHS was removed by two rounds of ethanol precipitation. Briefly, 10 µL of 5 M sodium chloride solution and 400 µL ice-cold 100% ethanol were added directly to the reaction mixture and incubated for 30 min at –30 °C. After centrifugation (14,000 g for 30 min at 4 °C) the supernatant was removed, and the pellet was dissolved in 100 µL of 1xPBS (pH 7.4). This procedure was repeated once and after centrifugation the pellet was washed with 95% ice-cold ethanol (v/v, in water). The mixture was centrifuged again (14,000 g for 15 min at 4 °C) and the pellet was lyophilized and stored at –30°C.

Preparation of eYFP-ssDNA conjugates

Conjugation reactions were performed on a 100 µL scale using 30 µM protein and 90 µM BCN-ssDNA in 100 mM potassium phosphate buffer, pH 7.2. The reaction mixture was incubated for 4 h at RT in the dark under continuous shaking at 800 rpm. Unreacted ssDNA and unreacted protein were removed by size exclusion column chromatography (Superdex 200 10/300 preparative column (GE Healthcare), eluent: 10 mM potassium phosphate buffer + 100 mM NaCl), and ion-exchange chromatography respectively. After equilibration of the ion-exchange column (0.5 mL strong anion-exchange spin columns, Thermo Scientific) with purification buffer (10 mM KPi, pH 7.0), the protein mixture was directly loaded onto the column in 400 µL fractions, according to the manufacturer's instructions. Elution was performed by a stepwise increase in NaCl concentration in the buffer (100 mM to 600 mM). eYFP-ssDNA conjugates eluted between 400 and 500 mM NaCl and were characterized by SDS-PAGE (Figure S1C). Elution fractions containing pure eYFP-ssDNA conjugates were pooled and the concentration was determined by measuring the absorption at 513 nm.

Preparation and characterization of ssDNA introduced-, and dye labeled HRP and Mb

Synthesis of Heme-azide

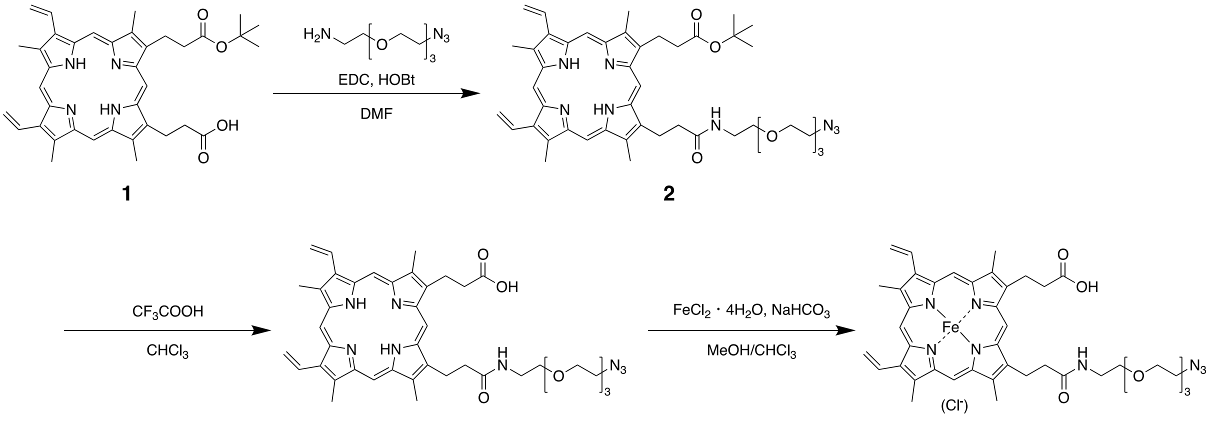

1 **2** **3** **Heme-azide**

Scheme S1. Synthesis of Heme-azide. Each protoporphyrin IX derivative contains a structural isomer due to the modified position at the 6- or 7-propionate side chain.

Porphyrin 2. Under an N_2 atmosphere, porphyrin **1** (31.0 mg, 50 μmol) was added to a solution of 1-(3-dimethylaminopropyl)-3-ethylcarbodiimide hydrochloride (EDC•HCl, 23.3 mg, 120 μmol) and 1-hydroxybenzotriazole (HOBt, 16.2 mg, 120 μmol) in dry *N*,*N*-dimethylformamide (DMF, 2.5 mL) on ice, followed by addition of a solution of 11-azido-3,6,9-trioxaundecan-1-amine (24 μL, 120 μmol) in dry DMF (1 mL). The mixture solution was heated to room temperature and stirred for 12 h. After the addition of $CHCl_3$ (30 mL), the organic layer was washed with 5% citric acid aqueous solution (3 times) and water (3 times), and then the solvent was removed *in vacuo*. The crude product was purified by silica gel chromatography ($CHCl_3$/MeOH = 99/1, v/v). The solvent was removed *in vacuo* to give porphyrin 2 as a brown solid (21.7 mg, 26.5 μmol, 53%). ^1H NMR (400 MHz, $CDCl_3$) δ: 10.22 (m, 4H) {the signals of the meso-protons split into several signals due to the presence of the regioisomer (Figure ill). The abundance ratio is 1:1 based on the peak intensities}, 8.34 (m, 2H), 6.42 (m, 2H), 6.24 (m, 2H), 6.11 (m, 1H), 4.44 (m, 4H), 3.77 (m, 6H), 3.67 (m, 6H), 3.21 (m, 6H), 3.05 (m, 2H), 2.95 (t, *J* = 5.2 Hz, 2H), 2.89 (m, 2H), 2,79 (m, 2H), 2.49 (m, 2H), 2.20 (m, 2H), 1.90 (m, 2H), 1.32 (s, 9H), -3.6 (s, 2H). ESI-TOF MS: *m*/*z* = 819.4548, calculated for $C_{46}H_{58}N_8O_6$ $[M + H]^+$ 819.4552.

Porphyrin 3. Porphyrin **2** (21.7 mg, 26.5 μmol) was dissolved in 22 mL of a $CHCl_3/CF_3COOH$ (1:1, v/v) solution. After stirring for 4 h, the solvent was evaporated. The product was precipitated in $Et_2O/CHCl_3$ to give porphyrin **3** as a brown solid (13.0 mg, 17 μmol, 64%). ^1H NMR (400 MHz, $CDCl_3$) δ: 10.19 (m, 4H) {the signals of the meso-protons split into several signals due to the presence of the regioisomer (Figure S11). The abundance ratio is 1:1 based on the peak intensities}, 8.29 (m, 2H), 6.98 (m, 1H), 6.39 (d, *J* = 17.6 Hz, 2H), 6.21 (d, *J* = 11.6 Hz, 2H), 4.43 (m, 4H), 3.67 (m, 6H), 3.64 (m, 6H), 3.28 (m, 4H), 3.17 (m, 2H), 3.02 (m, 4H), 2.96 (m, 2H), 2.83 (m, 2H), 2,73 (m, 2H), 2.39 (s, 2H), 2.15 (m, 2H), -3.69 (s, 2H). ESI-TOF MS: *m*/*z* = 785.3742, calculated for $C_{42}H_{50}N_8O_6$ $[M + Na]^+$ 785.3746.

Heme-azide. Porphyrin **3** (13.0 mg, 17.1 μmol), $FeCl_2$•$4H_2O$ (86.5 mg, 437 μmol), and $NaHCO_3$ (14.5 mg, 427 μmol) were dissolved in N_2 purged MeOH/$CHCl_3$ (1/2, v/v, 48 mL) under an N_2 atmosphere. After stirring at 50 ˚C for 4h, the solution was washed with 0.1 M aqueous HCl (6 times), dried over Na_2SO_4, and the solvent was evaporated. The residue was dissolved in a minimum volume of $CHCl_3$. The addition of n-hexane then caused precipitation of a brown solid. The solid was collected and dried to afford Heme-azide as a brown solid (7.7 mg, 9.0 μmol, 53%). ESI-TOF MS: *m*/*z* = 816.3040, calculated for $C_{42}H_{48}FeN_8O_6$ $[M – Cl]^+$ 816.3041.

Preparation of reconstituted HRP and Mb with Heme-azide

Removal of heme from horse radish peroxidase (HRP) and myoglobin (Mb) was performed using Teale's method as follows. A solution of proteins (50 μM as a monomer) in 100 mM of L-histidine solution at 4 ˚C was acidified to pH 2 by addition of 1 M aqueous HCl. Unbound heme was extracted with 2-butanone (5 times) and the colorless aqueous solution was neutralized by dialysis with 100 mM potassium phosphate buffer, pH 7.0, at 4˚C. A DMSO solution of heme-azide (2 mM, 200 μL) was added to a solution of obtained apoprotein (30 μM, 10 mL) in 100 mM potassium phosphate buffer, pH 7.0. After gently shaking overnight at 4 ˚C, excess heme-azide was removed with

a HiTrap desalting column. Concentrations of the obtained Mb and HRP were determined by measuring the absorption at 408 nm and 403 nm assuming an extinction coefficient of 1.8×10^5 M^{-1} cm^{-1} and 1.0×10^5 M^{-1} cm^{-1}, respectively.

Preparation of ssDNA and Dye introduced HRP and Mb

Conjugation reactions were performed on a 100 µL scale using 30 µM HRP-heme-azide or Mb-heme-azide and 90 µM BCN-ssDNA in 100 mM potassium phosphate buffer, pH 7.2. The reaction mixture was incubated for 4 h at RT, whilst shaking at 800 rpm. Unreacted ssDNA was removed by size exclusion column chromatography (Superdex 200 10/300 preparative column (GE Healthcare), eluent: 100 mM potassium phosphate buffer). Cy3-NHS and Cy5-NHS (1 µL, 10 mM stock solution in dry DMSO) were added to the protein solution (10 µM, 200 µL) and gently shaken overnight at 4 ˚C in the dark. After adding 2 eq of complementary uptake strand and incubating for 30 min at 4˚C, unreacted dye and excess uptake strand were removed by size exclusion column chromatography (Superdex 200 10/300 preparative column (GE Healthcare), eluent: 100 mM potassium phosphate buffer, Fig S2).

Formation of coacervate artificial cells containing protein-ssDNA conjugates

Quaternized amylose (Q-Amylose), carboxymethylated amylose (CM-Amylose), nitrilotriacetic acid modified amylose (NTA-amylose) and, the terpolymer poly(ethylene glycol)-poly-(caprolactone-g-trimethylene carbonate)-poly- (glutamic acid) (PEG-PCLgTMC-PGlu) were prepared in accordance with previously published procedures.[5],[6] For the preparation of coacervates, Q-amylose, CM-amylose and NTA-amylose were dissolved at a concentration of 1 mg/mL in PBS containing 5 mM $MgCl_2$, pH 7.4. Next, 25 µL CM-amylose and 50 µL PBS containing 5 mM $MgCl_2$, pH 7.4, were mixed in a Lo DNA-binding tube, and then 25 µL Q-amylose was added whilst shaking (1500 rpm). After 5 minutes, Terpolymer (PEG-PCLgTMC-PGlu) (2 µL, 25 mg/mL in DMSO) was added to the solution to stabilize the coacervates. For protein uptake, ssDNA-modified protein and a corresponding uptake strand were added to the coacervate solution at a final concentration of 100 nM (unless stated otherwise) and incubated 30 min at 4 ˚C. To trigger protein release, (non-) complementary releaser strands at a final concentration of 120 nM (unless stated otherwise) were added. Coacervates containing Ni^{2+} and NTA-amylose were prepared as previously described.[5] Briefly, 20 µL CM-amylose, 5 µL NTA-amylose, 3.75 µM $NiCl_2$ 50 µL PBS containing 5 mM $MgCl_2$ and were mixed in a Lo ssDNA-binding tube. To this shaking solution, 25 µL Q-amylose was added (1500 rpm). After 5 minutes, Terpolymer (2 µL, 25 mg/mL in DMSO) was added to the solution to stabilize the coacervates.

Confocal microscopy

Images were acquired using a Leica TCS SP8 confocal laser scanning microscope equipped with a white light laser, using a HCX PL APO CS 63.0x 1.20 NA water immersion objective and HyD detector. The pinhole was set to 1 Airy Unit (111 µm). Single plane images of 1024x1024 pixels were acquired with a scan rate of 600 Hz, and line averaged 4 times. The respective channels were recorded sequentially using the following parameters:
Yellow channel (eYFP): excitation at 489 nm; emission at 500-540 nm.
Magenta channel (Cy3): excitation at 570 nm; emission at 580-630 nm
Cyan channel (Cy5): excitation at 650 nm; emission at 670-750 nm

Image analysis

Confocal images were analyzed with FIJI (ImageJ). Fluorescence distribution between artificial cell populations was determined using standard ImageJ functions. Particles were manually selected with the *elliptical selection tool*, size and mean pixel intensity were determined with the *measure tool.* For each analysis >10 particles were included. For quantification, mean grey values were normalized to the maximum grey value of the image that was used for comparison (time point 0 for time measurements, or before addition of the releaser strand for release studies).

Supplementary figures and tables

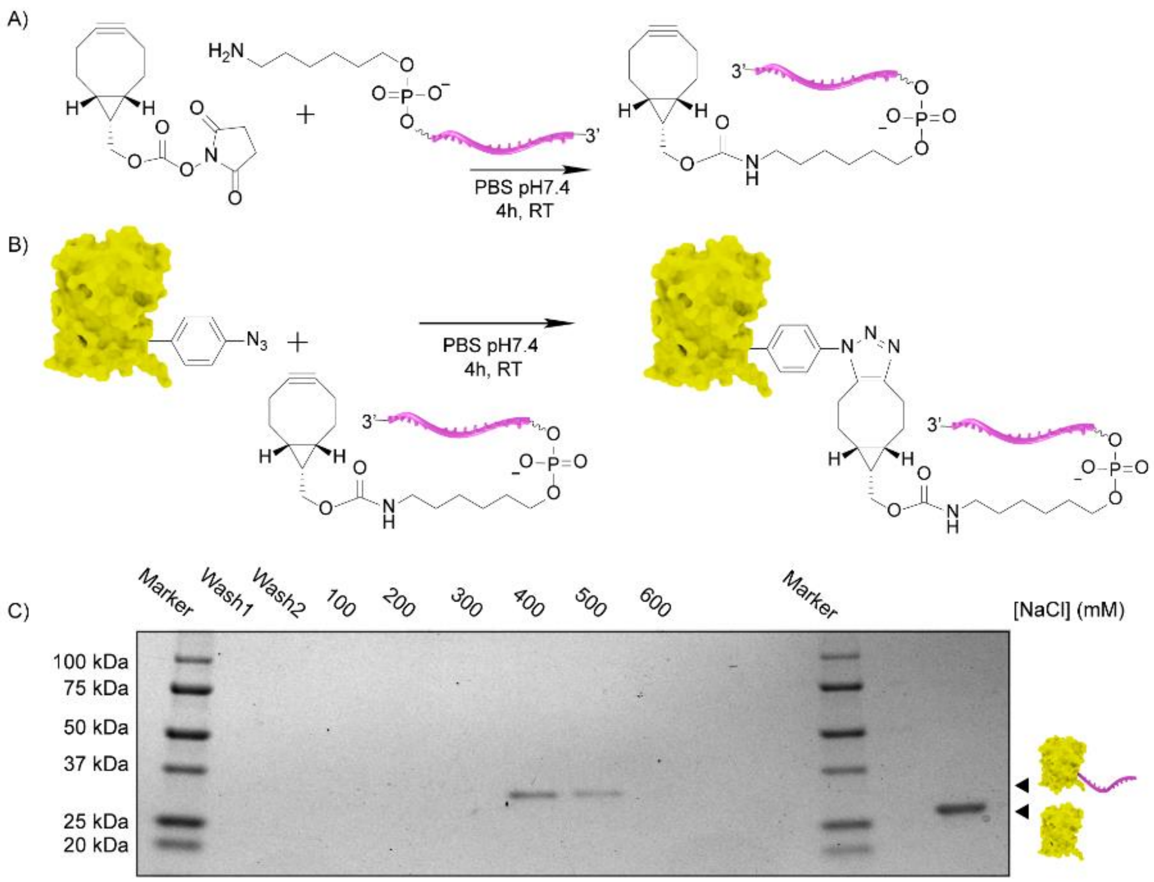

Figure S1. Conjugation of ssDNA to eYFP (pAzF) using strain-promoted alkyne-azide cycloaddition. A) Reaction scheme for the modification of 5'-amine modified C6 ssDNA with bicyclononyne- N-succinimidyl carbonate (BCN-NHS). B) Reaction scheme for the modification of eYFP (pAzF) with BCN modified ssDNA. Reactions were performed for 4h at room temperature (RT) in phosphate-buffered saline (PBS), pH7.4. C) SDS-PAGE analysis of the conjugation reaction of BCN-ssDNA (4.2 kDa) to eYFP (30.5 kDa) and purification by anion exchange chromatography. eYFP modified with ssDNA appeared as a band with a higher mass than eYFP and eluted between 400 and 500 mM NaCl.

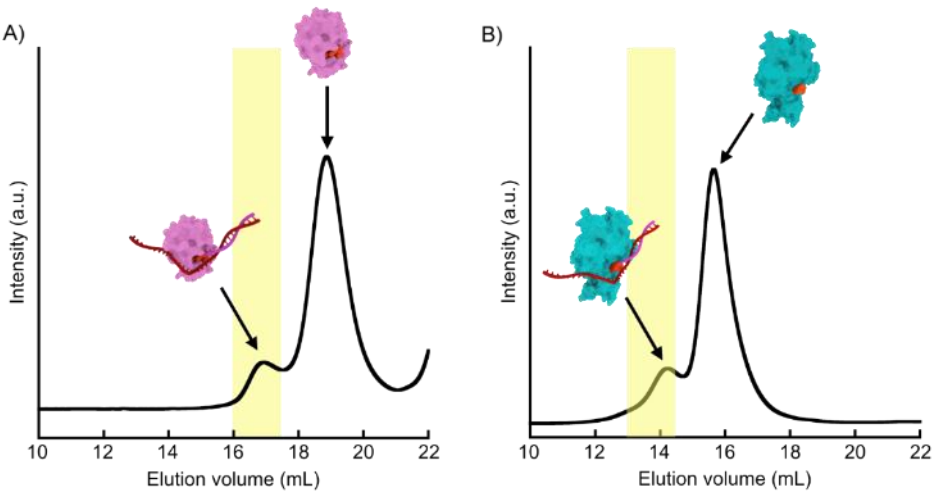

Figure S2. SEC traces of (A)) 12nt-ssDNA-Mb-Cy3 with UPT A-20 and (B) 12nt-ssDNA-HRP-Cy5 with UPT A-40, which were detected at 550 nm and 650 nm, respectively. The yellow area was collected as protein conjugate. Eluent is 100 mM potassium phosphate buffer at pH 7.0 and 4 ℃ at a flow rate of 0.5 mL min^{-1}.

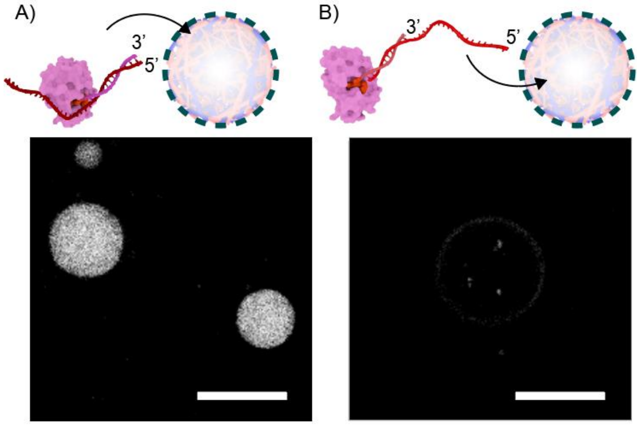

Figure S3. Schematic and confocal images of the partitioning of 12nt-ssDNA-Mb-Cy3 conjugates in coacervates within a time frame of 10 min. The complementary sequence of the uptake strand (UPT A-20) is located at (A) the 3' end or (B) the 5' end. The location of the non-complementary part of this uptake strand dictates the sequestration efficiency of Mb by the coacervates. Electrostatic interactions between the non-complementary ssDNA part and Mb are postulated to play a part in this difference in efficiency. When the non-complementary part is located 5' to the handle (A), the negative charge of the ssDNA part could shield some of the positive charges displayed on the surface of Mb, thereby neutralizing these charges, rendering the whole complex more anionic. When the non-complementary part is located 3' to the handle, such electrostatic interactions might be hindered (by the rigidity of the dsDNA, protruding away from the protein). After adding REL strand the solutions were incubated for 30 minutes at room temperature. The experiment was performed in PBS containing 5 mM MgCl2, pH 7.4, ionic strength (*I*) = 185 mM. Scale bars represent 20 µm.

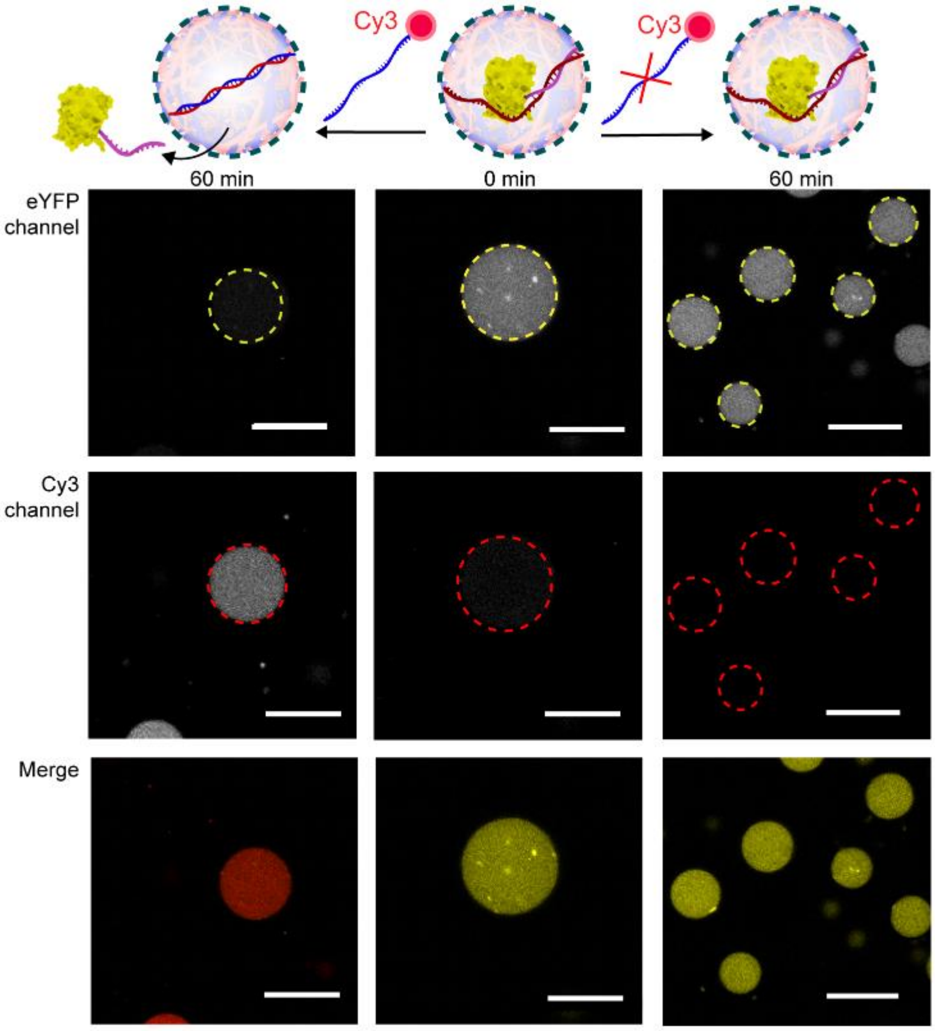

Figure S4. (A) Schematic and confocal images of the release or retention of 12nt-ssDNA from coacervate-based synthetic cells after 60 minutes with and without addition of the releaser strand (REL A-20, blue) respectively. The experiment starts at 0 min when eYFP is uptaken inside the coacervate core following hybridization with UPT A-20 (middle column). Addition of Cy3-labeled REL A-20 results in its sequestration and subsequent hybridization with UPT A-20. Following strand displacement, the release of 12nt-ssDNA-eYFP is triggered (left column). Without addition of REL A-20, 12nt-ssDNA-eYFP is retained in the coacervate-core (right column). Yellow is eYFP; Red is Cy3 labeled releaser strand. Scale bars represent 20 µm. After adding REL strands, the solutions were incubated for 60 minutes at room temperature. The experiment was performed in PBS containing 5 mM MgCl2, pH 7.4, ionic strength (*I*) = 185 mM.

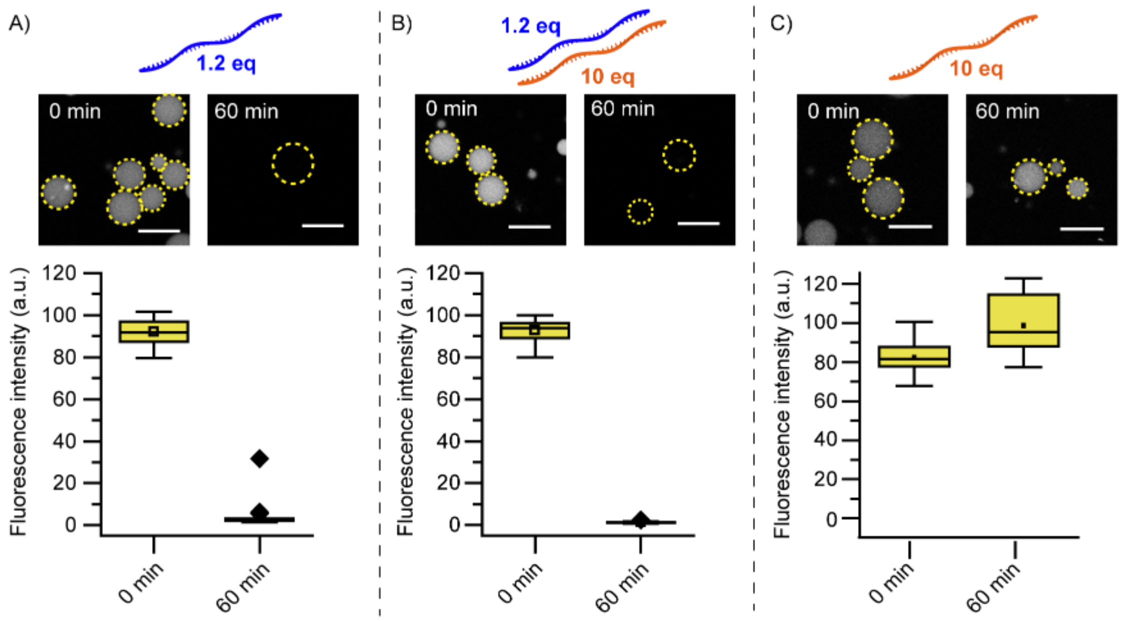

Figure S5. Confocal images and fluorescence quantification of the release or retention of 12nt-ssDNA-eYFP from coacervate-based artificial cells following addition of (A) 1.2 equivalents REL A-20 (blue), (B) 1.2 equivalents REL A-20 mixed with 10 equivalents REL B-20 (orange) and (C) 10 equivalents REL B-20. The experiment starts at 0 min when 12nt-ssDNA-eYFP is uptaken inside the coacervate core following hybridization with UPT A-20. 60 minutes after addition of REL A-20, the intensity inside the coacervates is measured again. Box plots depict the fluorescence intensity of eYFP inside the coacervate core at 0 minutes (before the addition of releaser strand) and 60 minutes after addition of releaser strand at room temperature. The experiment was performed in PBS containing 5 mM MgCl2, pH 7.4, ionic strength (*I*) = 185 mM. Scale bars represent 20 µm. ■ represents the mean, ♦ represents outliers. Fluorescence intensity was determined inside the core of ≥ 15 coacervates per experiment.

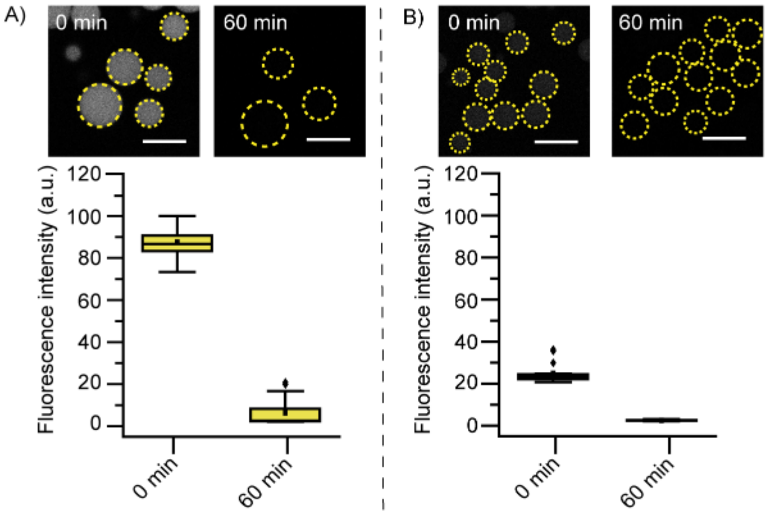

Figure S6. Confocal images and fluorescence quantification of the uptake of 12nt-ssDNA-eYFP in coacervate-based artificial cells and its release following addition of 1.2 equivalents REL A-20 in (A) PBS + 5 mM $MgCl_2$ and (B) PBS + 50 mM NaCl and 5 mM $MgCl_2$. The experiment starts at 0 min when 12nt-ssDNA-eYFP is uptaken inside the coacervate core following hybridization with UPT A-20. 60 minutes after addition of REL A-20, the intensity inside the coacervates is measured again. Box plots depict the fluorescence intensity of eYFP inside the coacervate core at 0 minutes (before the addition of releaser strand) and 60 minutes after addition of releaser strand. The experiment was performed in PBS containing 5 mM MgCl2, pH 7.4, ionic strength (*I*) = 185 mM. Scale bars represent 20 µm. ■ represents the mean, ♦ represents outliers. Fluorescence intensity was determined inside the core of ≥ 15 coacervates per experiment.

Table S6. Estimation of the net positive charge of the coacervate solution before and after addition of UPT3 strand. $[Q]^+$ was estimated by multiplying the concentration of Q-amylose in the sample with the degree of substitution (DS) of hydroxy groups replaced for a quaternary ammonium (Q), which equals 1. $[CM]^-$ was calculated by multiplying the concentration of CM-amylose with the DS of hydroxy groups replaced for a carboxymethyl (CM), which equals 0.4.

	Before UPT addition	After UPT3 addition
$[Q]^+$ in sample (µM)	545.3	545.3
$[CM]^-$ in sample (µM)	328.2	328.2
$[Nucleotide]^-$ (µM)	0	59.4
Net charge	+217.1	+157.7

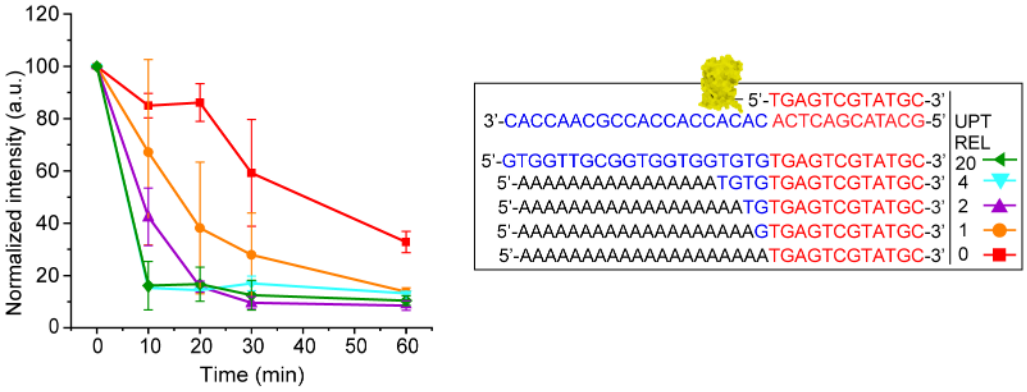

Figure S7. Reproducibility of the releasing speed of eYFP, dependent on releaser strand complementarity as shown in Figure 3B. Graph depicts the decrease in fluorescence intensity inside coacervates over time following the addition of releaser strands with complementary bases varying between 0+12 (0) and 10+12nt (20) at room temperature. For sequestration of eYFP with each UPT strand, all samples were incubated over 30 minutes at 4 ˚C before adding each REL strands. The experiment was performed in PBS containing 5 mM MgCl2, pH 7.4, ionic strength (*I*) = 185 mM. N=2, Fluorescence intensity was determined inside the core of ≥ 15 coacervates per experiment. Error bars represent standard deviation.

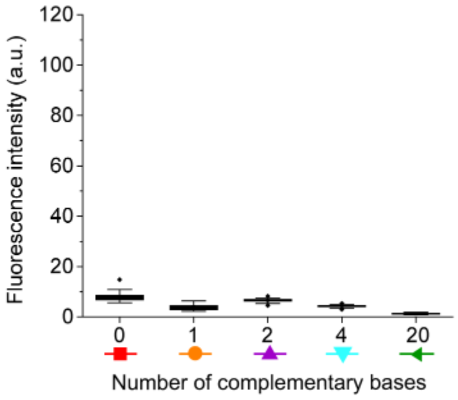

Figure S8. eYFP fluorescence intensity inside coacervate-based artificial cells after overnight incubation with releaser strands of different complementarity at 4˚C. Complementary bases in the releaser strand vary between 12+0nt (0) and 12+20nt (20). These box plots serve as an extension of the data depicted in Figure 3B. The experiment was performed in PBS containing 5 mM MgCl2, pH 7.4, ionic strength (*I*) = 185 mM. Fluorescence intensity was determined inside the core of ≥ 15 coacervates. Error bars represent standard deviation.

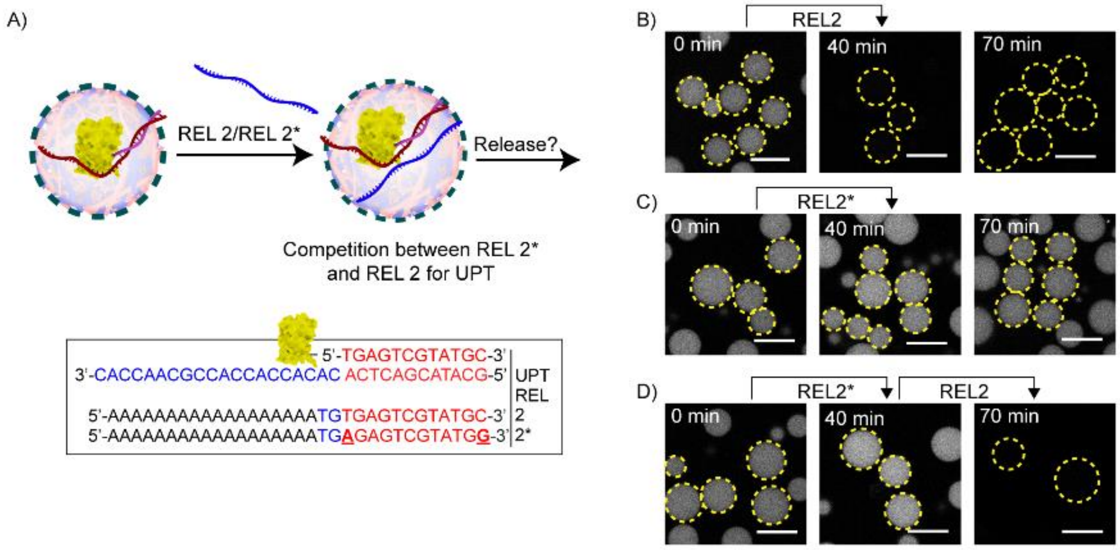

Figure S9. Release of 12nt-ssDNA-eYFP from coacervate-based artificial cells following addition of REL A-2 or REL A-2*. (A) Schematic illustrating the competition between REL A-2* and REL A-2 for hybridization with UPT A-20. Hybridization of REL A-2* with UPT A-20 could result in release of 12nt-ssDNA-eYFP, in a similar fashion as REL A-2. On the other hand, if REL A-2* does not hybridize with UPT, due to the incorporation of 2 mutations, no release would follow and REL A-2 would outcompete REL A-2*, finally resulting in release. (B) Confocal images showing the release of 12nt-ssDNA-eYFP from coacervates following REL A-2 addition. (C) Confocal images showing the absence of release of 12nt-ssDNA-eYFP from coacervates following REL A-2* addition within a time frame of 70 minutes. (D) Confocal images showing the absence of release of 12nt-ssDNA-eYFP from coacervates following REL A-2* addition, and subsequent release after REL A-2 addition. The melting temperatures of UPT A-20/ REL A-2 and UPT A-20/ REL A-2* are 52.9 ˚C and 43.9 ˚C, respectively (calculated with DINAMelt). Scale bars represent 20 µm. After adding the first and second REL strands, the solutions were incubated for 40 and 30 minutes, respectively, at room temperature. The experiment was performed in PBS containing 5 mM MgCl2, pH 7.4, ionic strength (*I*) = 185 mM.

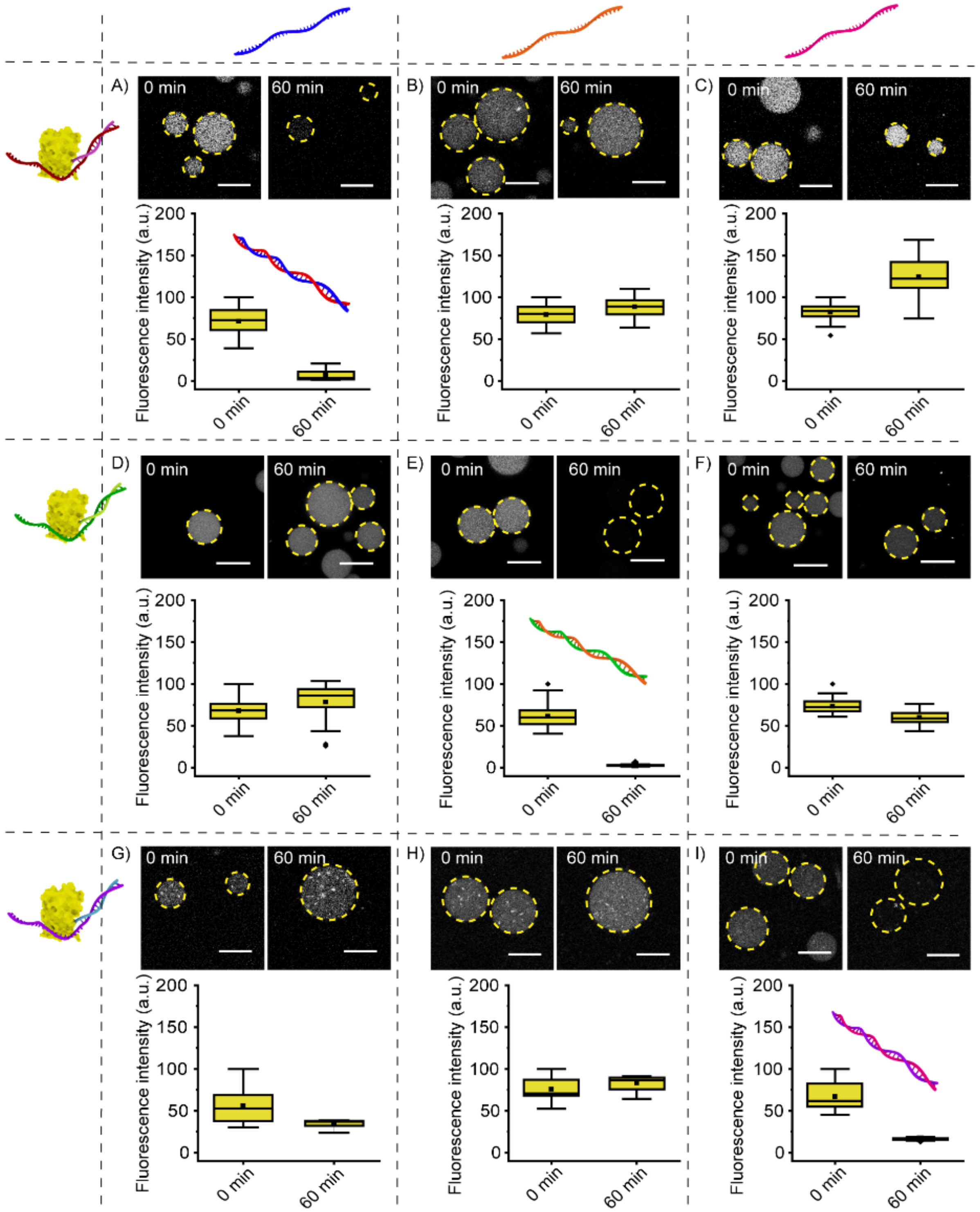

Figure S10. Schematic and confocal images with corresponding fluorescence quantification showing the release of 12nt-ssDNA-eYFP from coacervate-based artificial cells, only following addition of a releaser strand complementary to the uptake strand. When a mismatch releaser strand is added, 12nt-ssDNA-eYFP is retained in the coacervate phase. 12nt-ssDNA-eYFP with three different handle/UPT 20 strand combinations (from top to bottom: handle a/UPT A-20 (light purple/red); handle b/UPT B-20 (light green/dark green); and handle c/UPT C-20 (light blue/purple) are displayed in the first column. The different complementary releasing strands are displayed in the first row (from left to right: REL A-20 (dark blue); REL B-20 (orange); and REL C-20 (pink). Uptake/releaser (UPT 20/REL 20) strand pairs are represented by the double strand helix (form upper left to lower right: red/dark blue; light green/orange; and purple/pink). A) Release of 12nt-ssDNA-eYFP (handle a) after 60 minutes following addition of a complementary REL A-20 strand. B) Retention of 12nt-ssDNA-eYFP (handle a) following addition of a non-complementary REL B-20 strand. C) Retention of 12nt-ssDNA-eYFP (handle a) following addition of a non-

complementary REL C-20 strand. D) Retention of 12nt-ssDNA-eYFP (handle b) following addition of a non-complementary REL A-20 strand. E) Release of 12nt-ssDNA-eYFP (handle b) following addition of a complementary REL B-20 strand. F) Retention of 12nt-ssDNA-eYFP (handle b) following addition of a non-complementary REL C-20 strand. G) Retention of 12nt-ssDNA-eYFP (handle c) following addition of a non-complementary REL A-20 strand. H) Retention of 12nt-ssDNA-eYFP (handle c) following addition of a non-complementary REL B-20 strand. I) Release of 12nt-ssDNA-eYFP (handle c) following addition of a complementary REL C-20 strand. The experiment was performed in PBS containing 5 mM MgCl2, pH 7.4, ionic strength (*I*) = 185 mM. Scale bars represent 20 µm. Box plots depict the fluorescence intensity of eYFP inside the coacervate core at 0 minutes (before the addition of releaser strand) and 60 minutes after addition of releaser strand. ■ represents the mean, ♦ represents outliers. Fluorescence intensity was determined inside the core of ≥ 15 coacervates per experiment.

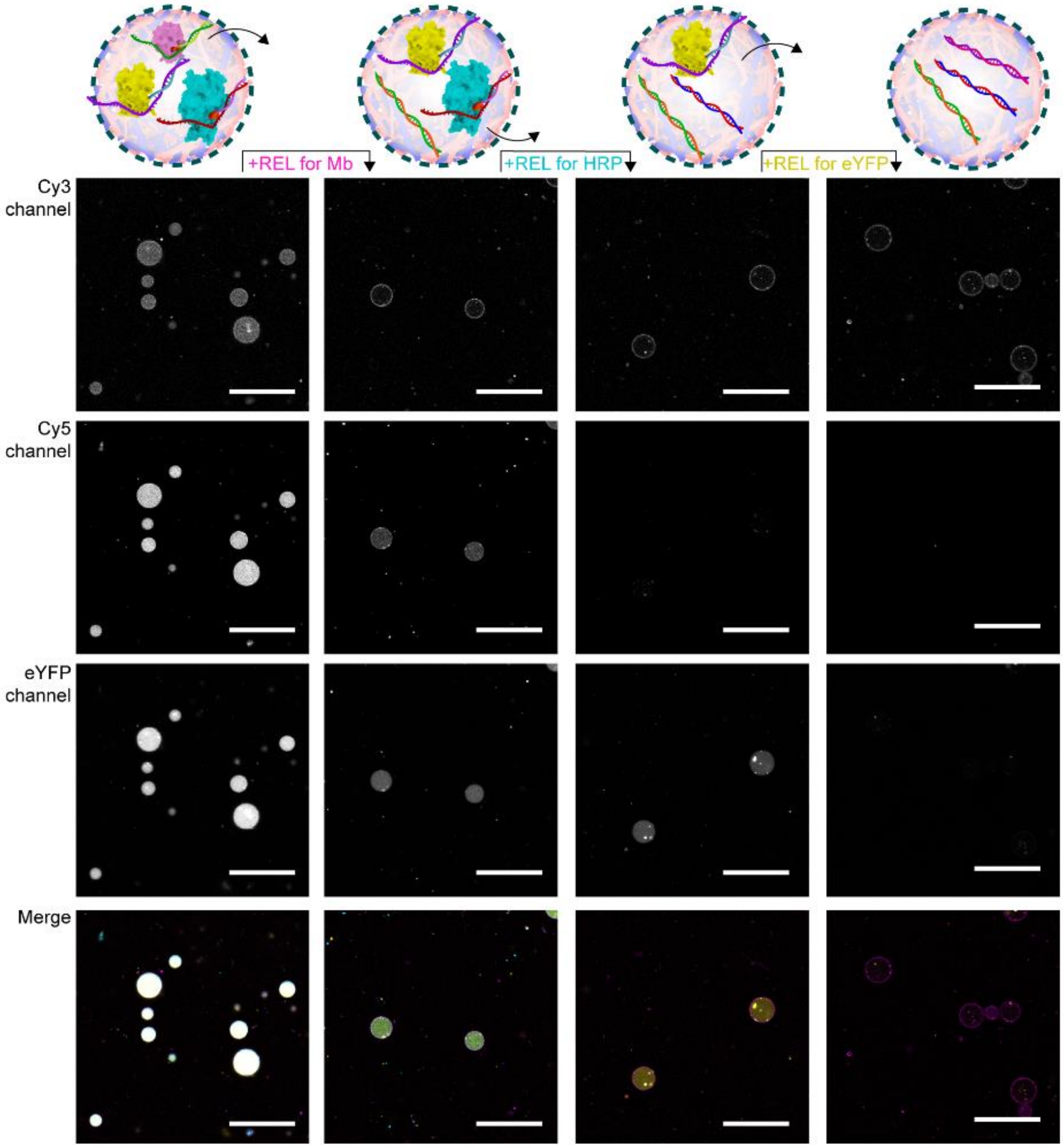

Figure S11. Schematic and confocal overview images showing the selective release of 12nt-ssDNA-Mb-Cy3, 12nt-ssDNA-HRP-Cy5 and 12nt-ssDNA-eYFP following addition of corresponding releaser (REL) strands. The experiment starts when 12nt-ssDNA-Mb, 12nt-ssDNA-HRP and 12nt-ssDNA-eYFP are co-sequestered inside the coacervates following hybridization with their corresponding complementary uptake strand (left column). The 12nt-ssDNA handle b conjugated to Mb hybridized with UPT B-20 (light green/dark green), 12nt-ssDNA handle a conjugated to HRP hybridized with UPT A-40 (light purple/red), and 12nt-ssDNA handle c conjugated to eYFP hybridized with UPT C-20 (light blue/purple). First REL B-20 (orange) was added (second column) and 12nt-ssDNA-Mb was released, then REL A-40 (dark blue) was added and 12nt-ssDNA-HRP was released (third column). Finally, REL C-20 (pink) was added and 12nt-ssDNA-eYFP was released (right column). Incubation time after adding each REL strands was 30 minutes. The experiment was performed in PBS containing 5 mM $MgCl_2$, pH 7.4, I = 185 mM. Yellow is eYFP; Pink is Cy3; and Cyan is Cy5. Scale bars represent 20 µm.

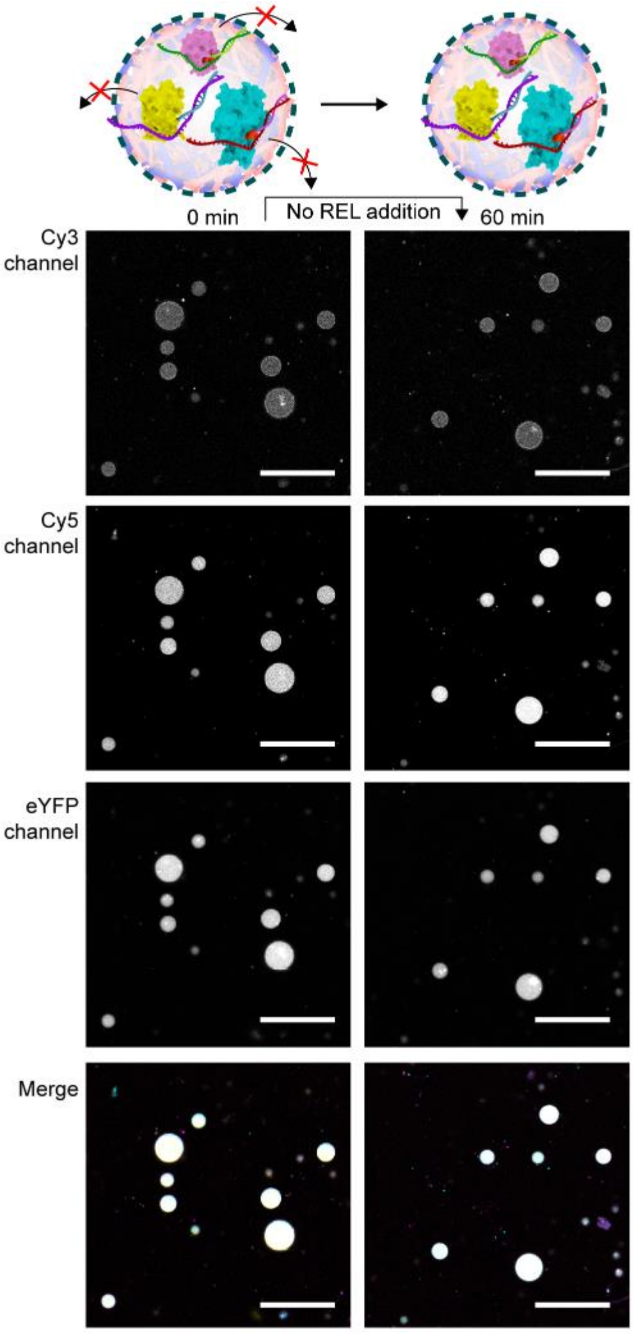

Figure S12. Schematic and confocal overview images showing retention of 12nt-ssDNA-Mb-Cy3, 12nt-ssDNA-HRP-Cy5 and 12nt-ssDNA-eYFP inside the coacervates after 60 minutes when omitting all corresponding releaser strands. The experiment starts when 12nt-ssDNA-Mb, 12nt-ssDNA-HRP and 12nt-ssDNA-eYFP are co-sequestered inside the coacervates following hybridization with a complementary uptake strand (left column). The 12nt-ssDNA handle b conjugated to Mb hybridized with UPT B-20 (light green/dark green), 12nt-ssDNA handle a conjugated to HRP hybridized with UPT A-40 (light purple/red), and 12nt-ssDNA handle c conjugated to eYFP hybridized with UPT C-20 (light blue/purple). After 60 minutes incubation in the absence of releaser strands at room temperature all the proteins remain inside the coacervate core (right). The experiment was performed in PBS containing 5 mM $MgCl_2$, pH 7.4, I = 185 mM. Yellow is eYFP; Pink is Cy3; and Cyan is Cy5. Scale bars represent 20 µm.

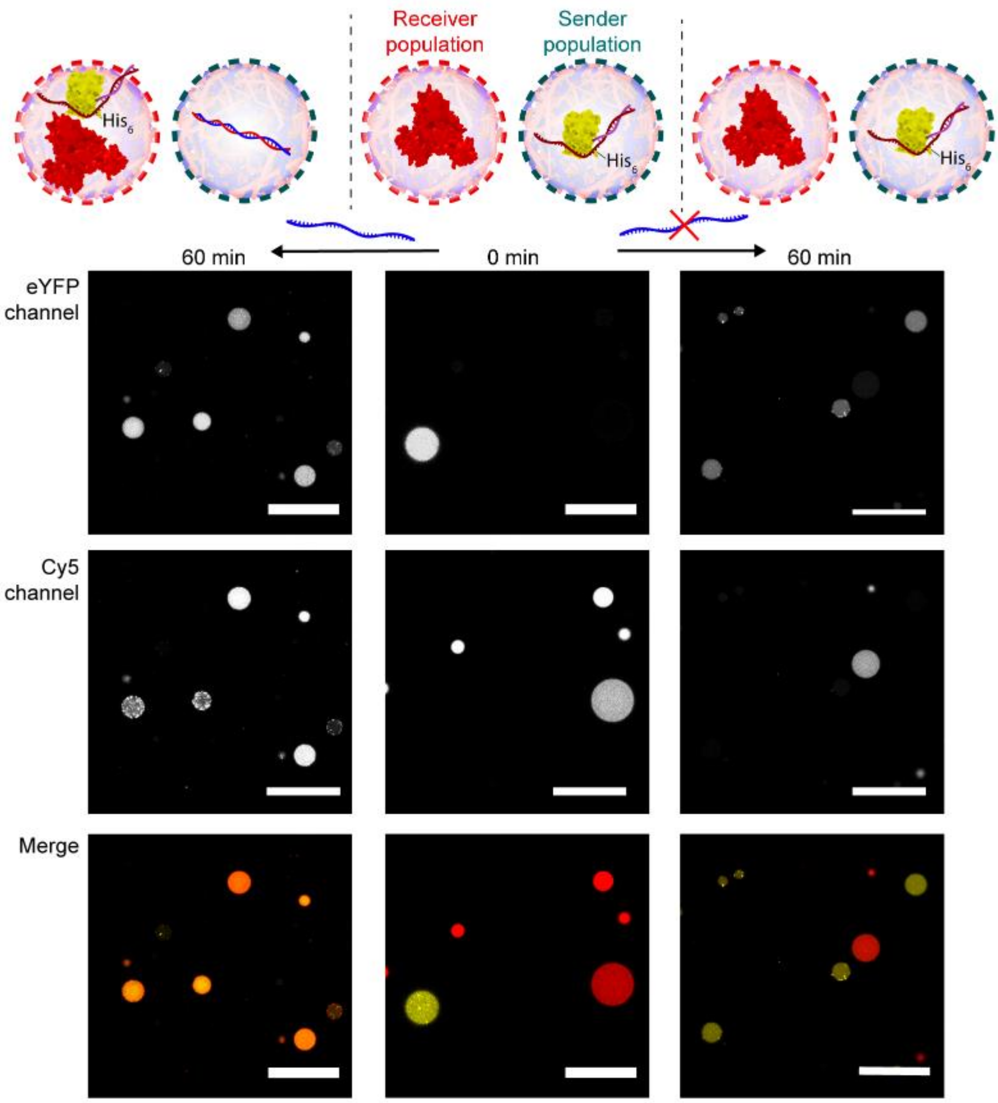

Figure S13. Schematic and confocal images showing the presence and absence of exchange between two artificial cell populations with or without addition of the releaser strand REL A-20 (blue). The sender population was loaded with a histidine-tagged 12nt-ssDNA-eYFP and uptake strand (UPT A-20), the receiver population was equipped with NTA-amylose, $Ni^{2+}$and Cy5 labeled succinylated BSA (Cy5-BSA). The experiment starts at 0 min when 12nt-ssDNA-eYFP is uptaken inside the coacervate core following hybridization with UPT A-20 (middle column). Addition of the releaser strand (REL A-20) triggers the release of 12nt-ssDNA-eYFP from the sender population. Subsequentially, 12nt-ssDNA-eYFP is sequestered by the receiver population via complex formation between the Histidine tag and Ni^{2+}/NTA-amylose (left column). After 60 minutes incubation at room temperature in the absence of releaser stand, no exchange of 12nt-ssDNA-eYFP from the sender to the receiver population has occurred (right column). The experiment was performed in PBS containing 5 mM MgCl2 and 3.75 µM NiCl2, pH 7.4, I = 185 mM. Yellow is eYFP; Red is succinylated Cy5-BSA. Scale bars represent 20 µm.

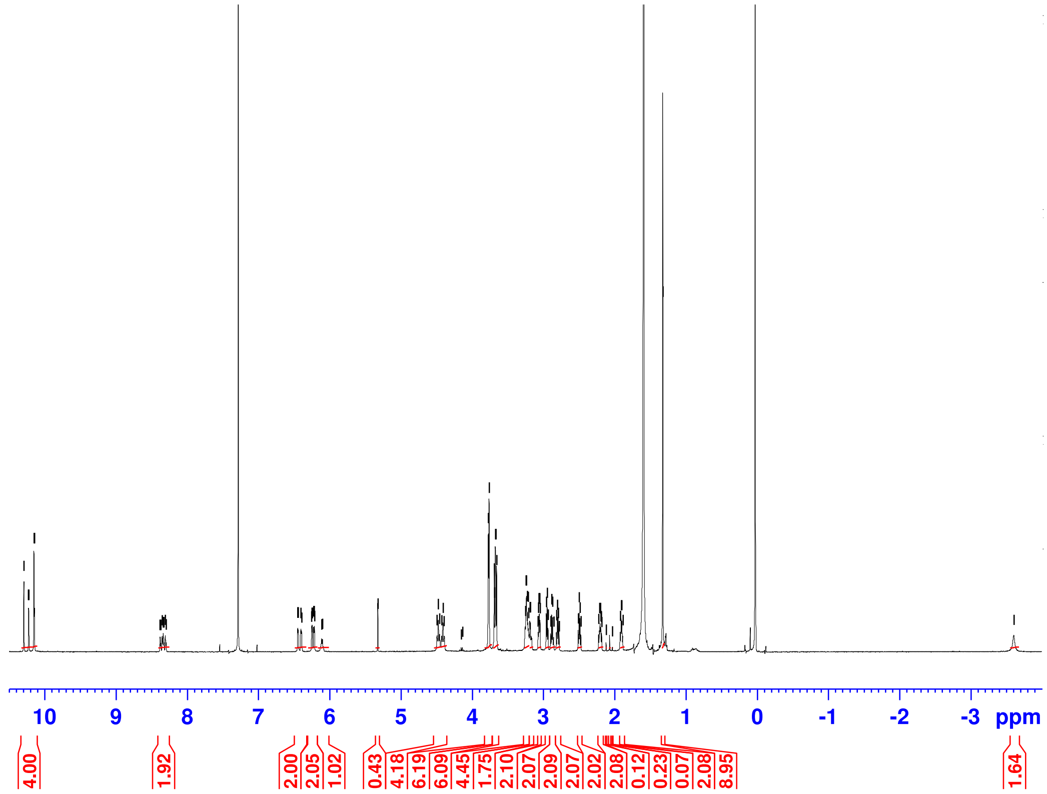

Figure S14. ^{1}H NMR spectrum (400 MHz, $CDCl_3$) of porphyrin **2**.

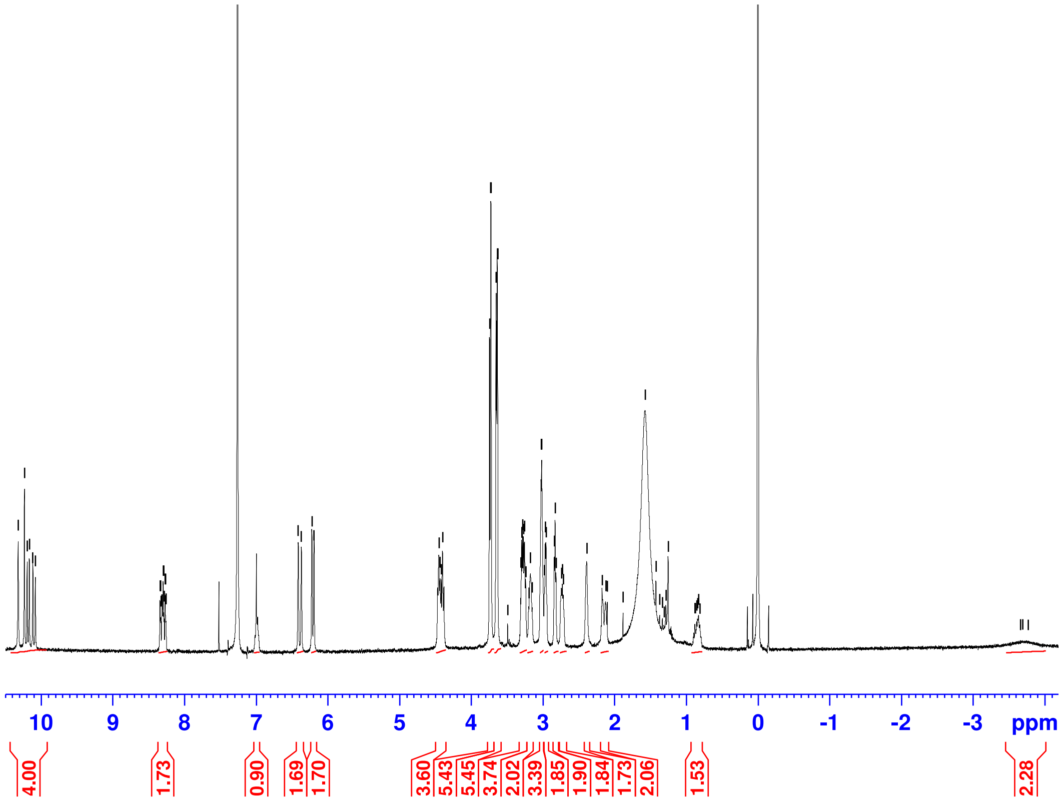

Figure S15. ^{1}H NMR spectrum (400 MHz, $CDCl_3$) of porphyrin **3**.

Supplementary references:

[1] T. Matsuo, T. Hayashi, Y. Hisaeda, *J. Am. Chem. Soc.* **2002**, *124*, 11234–11235.
[2] N. R.Markham, M. Zuker, *Nucleic Acids Res.*, **2005**, *33*, W577–W581.
[3] T. S. Young, I. Ahmad, J. A. Yin, P. G. Schultz, *J. Mol. Biol.* **2010**, *395*, 361–374.
[4] L. Wang, P. G. Schultz, *Chem. Biol.* **2001**, *8*, 883–890.
[5] W. J. Altenburg, N. A. Yewdall, D. F. M. Vervoort, M. H. M. E. van Stevendaal, A. F. Mason, J. C. M. van Hest, *Nat. Commun.* **2020**, DOI https://doi.org/10.1038/s41467-020-20124-0.
[6] A. F. Mason, B. C. Buddingh', D. S. Williams, J. C. M. van Hest, *J. Am. Chem. Soc.* **2017**, *139*, 17309–17312.
